# Frontiers from Radiomics in Molecular Imaging

**DOI:** 10.1155/2019/7919545

**Published:** 2019-01-02

**Authors:** Isabella Castiglioni, Francesca Gallivanone, Claudio Losio

**Affiliations:** ^1^Institute of Molecular Bioimaging and Physiology, Milan, Italy; ^2^San Raffaele Scientific Institute, Milan, Italy

The area of cancer research is nowadays rapidly evolving with basic research deepening on the understanding of molecular mechanisms underlying carcinogenesis and cancer cells spreading. A body of evidence showed that human cancers frequently display intratumor phenotypic heterogeneity whose nature can have profound implications for both tumor development and therapeutic outcomes. Genotypic and phenotypic profiles have shown increasing diagnostic and prognostic accuracy of ex vivo biopsy studies in several cancer diseases.

Recently, in vivo molecular imaging techniques, such as computerized tomography (CT), magnetic resonance imaging (MRI), functional diffusion-weighted imaging (DWI) MR, and positron emission tomography (PET), are showing intriguing results in characterizing lesions and predicting prognosis and therapy response in many cancer diseases, in particular when quantitative indexes of tumor are used, such as tumor functional volume, apparent diffusion coefficient, standardized uptake value, or other derived indexes. However, limited and contradictory results have been reported, and many authors argued that such macroscopic features are not able to properly reflect the intratumor heterogeneity responsible for the different progression or therapy response. Radiomics refers to mathematical methods used to extract a high number of descriptors from in vivo medical images of cancer. The basis hypothesis is that such descriptors are able to capture the heterogeneity of cells underlying the cancer genotype and phenotype.

We have invited authors to contribute with original research articles that could illustrate and stimulate the increasing effort to understand the heterogeneity of cancer phenotypes and to exploit the use of radiomics in targeted molecular imaging studies for the identification of diagnostic/predictive biomarkers of cancer.

In two of the five published papers, the authors focused on key issues at the basis of radiomic methodology, such as the need to extract and select radiomic features that are stable with respect to the whole-image processing procedures prior to their use as candidate biomarkers of diseases. Indeed, different experimental conditions, typically present in multicentre studies, e.g., different acquisition, reconstruction, and segmentation methods of lesion volume, can cause variations in radiomic features that must not be interpreted of biological significance. Consistently with these findings, both papers have confirmed the variability of radiomic descriptors, for MRI in the choice of the pulse sequence and for PET in the choice of the segmentation method.

It is worth noting that the two papers validated such variability on two innovative anthropomorphic phantoms. The use of phantoms has the principal advantage of the existence of a known ground truth for the assessment of radiomic features. More specifically, a 3D realistic digital MRI phantom of the brain was realized in the first paper with a simulation package tailored for fast generation of MRI with different sequences. Images with T1 and T2 weightings commonly used for 3T clinical brain MRI were provided. In the second paper, anthropomorphic lesions were realized with a current-generation 3D printer once extracted from PET images of real oncological lesions and ad hoc preprocessed to be compliant with the printer. The lesions were inserted in an anthropomorphic thorax phantom, thus mimicking real clinical situations typically presenting cancer primitive lesions with irregular shape and nonuniform radiotracer uptake.

The other three papers presented radiomic studies in real clinical settings, in head and neck cancer and in breast cancer. As general observation, a reduced feature set of no more than twenty-five radiomic traits was selected in all the studies, and this was a correct choice considering the limited number of clinical samples. However, each individual feature could only explain a small amount of variation in the outcomes. In the head and neck study, radiomic profiling was found to be a predictor of treatment failure in patients treated with concurrent chemoradiation therapy, with performance superior to the clinical assessment. In the breast cancer study, MRI radiomic descriptors including dynamic parameters were found to be predictive of nonresponse to neoadjuvant chemotherapy and able to differentiate between subtypes in women affected by locally advanced or invasive breast cancer.

These results, although obtained in retrospective single-centre studies, are consistent with previous findings and confirm the expected potential of radiomics in impacting patients for their personalized therapeutic decision.

